# Gene expression of protein synthesis, immunity and brain pathways specifically altered in Anorexia Nervosa

**DOI:** 10.1192/j.eurpsy.2024.192

**Published:** 2024-08-27

**Authors:** N. Ramoz, C. Verebi, N. Lebrun, P. Duriez, P. Gorwood, T. Bienvenu

**Affiliations:** ^1^INSERM U1266; ^2^CMME; ^3^GHU Paris Psychiatrie et Neurosciences, Paris, France

## Abstract

**Introduction:**

Anorexia nervosa (AN) is a severe and chronic psychiatric disorder, resulting from a voluntary food restriction, vomiting, use of laxatives and excessive exercises, leading in dramatic weight loss and high mortality. AN is a multifactorial disease involving genetic and epigenetic factors supporting that AN is a metabo-psychiatric disorder. The molecular mechanisms involved in the etiology of AN remain unclear. One work reported gene expression by RNA sequencing in peripheral blood before and after weight restoration in 6 AN patients (Kim 2013), and one RNA sequencing in human iiPSC-derived neurons from 4 patients and 4 controls (Negraes 2017). To date, the profile of expression of genes and proteins in AN is undetermined.

**Objectives:**

In this study, our goal is to identify specific gene expression signatures from circulating blood nuclear cells to decipher the pathophysiology of AN and characterize biomarkers that can be used for diagnostic or prognostic of AN.

**Methods:**

All consented participants are recruited at Sainte-Anne Hospital, Paris, France, using DSM5 criteria. They had a blood draw in Paxgene tube for the collection of RNAs. Total RNA was extracted from peripheral blood mononuclear cells of 15 patients suffering of AN and 15 healthy controls. All messenger RNAs are sequenced on a Novaseq plateform. Reads are aligned to the human genome 19 and statistical analyses on the read counts for differentially expressed genes are computed with DESeq2.

**Results:**

The total RNA sequencing allows us to identify 673 dysregulates genes (p adjusted value <0.01, fold change >1,5). Among them, 248 are down-regulated and 425 are up-regulated genes in AN patients compared to controls. From them, 151 transcripts are annotated as pseudogene and 45 are referenced as antisense RNA. Of the 522 remaining transcripts, 424 correspond to a transcript or protein annotated by HGNC and ENSEMBL and 93 are known pseudogenes. A large number of proteins resulting from the expression of deregulated genes interact with each other and form a statistically enriched network impacting biological processes. They are mainly increased and acting in the cellular machinery allowing protein synthesis (biological process: transcription, ribosome, spliceosome and mitochondria). In contrast, down-regulated genes present an enrichment in genes involved in immunity pathways. Finally, several genes are also expressed in the brain. We observed a significant enrichment of genes expressed in the blood and brain tissues.

**Conclusions:**

We identify specific profiles of gene expression in AN. Several genes are both blood and brain tissue expression. Some genes are good candidates for biomarker of the diagnostic in AN that need to be investigated in a longitudinal study to evaluate their useful as prognostic biomarker of AN.

This work is supported by Fondation de France & Fédération Recherche sur le Cerveau.

**Disclosure of Interest:**

None Declared

